# Biochemical Analysis of Organic Acids and Soluble Sugars in Wild and Cultivated Pomegranate Germplasm Based in Pakistan

**DOI:** 10.3390/plants9040493

**Published:** 2020-04-11

**Authors:** Muhammad Nafees, Muhammad Jafar Jaskani, Ishtiaq Ahmad, Maryam, Irfan Ashraf, Ambreen Maqsood, Sunny Ahmar, Muhammad Azam, Sajjad Hussain, Asma Hanif, Jen-Tsung Chen

**Affiliations:** 1 Department of Horticultural Sciences, University College of Agriculture and Environmental Sciences, The Islamia University of Bahawalpur, Bahawalpur 63100, Pakistan; 2 Institute of Horticultural Sciences, University of Agriculture, Faisalabad, Faisalabad 38040, Pakistan; jjaskani@uaf.edu.pk (M.J.J.); muhammad.azam@uaf.edu.pk (M.A.); 3 Department of Botany, the Government Sadiq College Women University, Bahawalpur 63100, PakistanAsma_hanif@gmail.com (A.H.); 4 Department of Forestry, Range and Wildlife Manangement, University College of Agriculture and Environmental Sciences, The Islamia University of Bahawalpur, Bahawalpur 63100, Pakistan; Irfan.ashraf@iub.edu.pk; 5 Department of Plant Pathology, University College of Agriculture and Environmental Sciences, The Islamia University of Bahawalpur, Bahawalpur 63100, Pakistan; ambreen.maqsood@iub.edu.pk; 6 Guangxi Key Laboratory of Agric-Environment and Agric-products Safety, Agricultural College of Guangxi University, Nanning 530004, China; 7 National Key Laboratory of Crop Improvement Genetics, College of Plant Sciences and Technology, Huazhong Agricultural University, Wuhan 430070, China; sunny.ahmar@yahoo.com; 8 Department of Horticulture, Bahauddin Zakariya University Multan, Multan 60000, Pakistan; sajjad.hussain@bzu.edu.pk; 9 Department of Life Sciences, National University of Kaohsiung, Kaohsiung 811, Taiwan

**Keywords:** pomegranate germplasm, characterization, HPLC, organic acids, sugar contents, fruit weight

## Abstract

Pomegranate is famous for its health benefiting chemical and biochemical constituent compounds. The present study was undertaken to characterize pomegranate germplasm for its various fruit traits, acids, and sugar profiling through high performance liquid chromatography (HPLC) analysis. Among 11 detected acids and 8 sugars, citric acid and fructose were predominant in 18 domestic and 5 wild genotypes, respectively. Fruit weight, aril weight and wood portion index (WPI) were ranged from 15.82% to 24.42%, 10.99% to 113.78%, and 2.39% to 17.25%, respectively. Genotypes were grouped as sweet, sweet–sour, sour–sweet, and sour based on citric acid contents. Lactic acid and pyruvic acid showed the highest correlation (*r* = 0.92), however, sour and sweet genotypes had strong association for acids and sugars, respectively. Straddling of dendrogram showed the flow of genetic material in a cultivated location with wild and cultivated pomegranates grouped in different classes, however, wild and sour landraces grouped in the same class with 71% similarity of traits. Based on the observations of the current study, it was concluded that selected wild and arid zones (Multan, Bahawalpur) genotypes are poor in nutrients (acid and sugars) quality, however, genotypes of Rahim-Yar-Khan, Muzafar Garh, and Khyber Pakhtunkhwa have a better composition of sugars and acids.

## 1. Introduction

An overwhelming number of reports have shown that plant-based foods contain an array of health-enhancing compounds that helps to maintain human health and contribute to food security. Apart from other traditional nutrient elements over a dozen naturally bioactive phytochemicals have been identified, the majority of which constitutes horticultural plant origin [1,2,3]. Pomegranate is one such round (typically yellow) fruit of immense horticultural significance because of its health-promoting biochemical phyto-nutritional components [4,5,6]. Recent scientific findings verify traditional usage of pomegranate as a medical remedy and indicate that its fruit, flower parts, plant bark, and leaves contain bioactive phytochemicals that are antimicrobial, reduce blood pressure [7], polyphenols in pomegranate are beneficial for dengue fever, diabetic, and cancer patients [8]. Fruit demand has been increased due to public awareness regarding its benefits, and the scientific community is emphasizing research programs for its improvement. [9]. Prominent pomegranate cultivars in the world are red in fruit and aril color with more juice percentage [10]. They have good fruit size with less wood portion index and wide supply window. Pakistan has undersized fruit with less juice percentage and narrow supply window. However, we also have white to pinkish-white aril color pomegranate fruit with diverse total soluble solids (TSS), phenolic, and antioxidant contents [11] showing a diverse genetic base in Pakistani pomegranates.

Soluble sugars in fruits of wild and cultivated pomegranates ranged from 17.57 to 19.99 mg/100 g and 13.13 to 16.55 mg/100 g of fruit with a high level of glucose and fructose, respectively [10]. Moreover, there is no galactose with traces of maltose in pomegranate aril juice [12] and a high range of reducing sugars and TSS (13.89 and 29.83 g/100 mL, 15.17 to 22.03 °Brix, respectively) in cultivated pomegranates [13]. Tezcan et al. [14] recorded fructose and glucose as the main source of energy and sweetness in pomegranate, moreover, the taste of pomegranate fruit juice is determined through the level of organic acids and sugar contents [10]. They reported that Tunisian cultivars contained more sugars than Spanish with the highest fructose and glucose (82.2 and 78.0 g/L), respectively. Spanish, Turkish, and Tunisian pomegranate cultivars contain high fructose than glucose, however, the latter was high in Russian, Saudi Arabian, and Turkish pomegranate cultivars [15,16,17]. There was a low level of sucrose, arabinose, and traces of galactose; and no sorbitol and maltose were detected in Tunisian pomegranates [10].

Pomegranate accessions of Iran and Spain had a high concentration of acetic and fumaric acids [16] whereas Tunisian cultivars lack these acids [10]. A high level of ascorbic acid was detected in sour pomegranates with traces of tartaric acid whereas sweet cultivars were restricted with more oxalic acid in Turkish pomegranates [10]. Citric and malic acids were major organic acids in sour and sweet cultivars, respectively [10,16,17,18]. There are various organic acids in pomegranate fruit juice but malic and citric acids are main and strongly correlated with sourness [19]. Melgarejo et al. [16] reported that sensory attributes in pomegranate and other fruit juices are mainly controlled by the combined effect of quality and quantity of organic acids, sugars, and anthocyanin. There were six detected organic acids in Tunisian pomegranate aril juice including malic, citric, succinic, oxalic, tartaric, and ascorbic acids with respective concentrations of 50%, 23%, 17%, 7%, 2%, and 0.3% of total organic acids [10]. In contrast to Tunisian pomegranate cultivars, citric acid was the major acid in fruit juice of Turkish pomegranates [17]. Moreover, pomegranate cultivars of Tunisia and Assaria cultivars of Portugal had a high level of oxalic compare to Spanish pomegranates [20]. Hasnaoui and coworkers [10] detected traces of fumaric acid and no acetic acid; however, these acids are quite different from Iranian [21] and Spanish [16] pomegranate cultivars. The concentration of citric, malic, and succinic acids were high in sour pomegranates, which showed a correlation of these acids with sourness [10], moreover, they proved that sourness in pomegranate fruits was negatively correlated with tartaric acid, however, a high correlation was observed between titratable acidity and citric acid. They also stated that the sourness index (SI) is considered reliable criteria in the characterization of pomegranate as sweet and sour accessions with SI ≥ 60 and < 10, respectively.

The morphochemical characterization is helpful in the identification of elite genotypes [10] and necessary to develop a thorough understanding of variability in crops for selection in the fresh and process industry. Therefore, morphological, biochemical, and genetic studies provide information that helps in a variety improvement program [17,22]. Countries like Spain, Iran, India, Israel, USA, and China, are currently engaged with pomegranate germplasm for identification, characterization, and conservation [18,19,20,21,22,23] and thus producing better quality pomegranate fruits under diverse climatic conditions.

The dissemination of pomegranate in India, Afghanistan, and Pakistan is believed to form its primary origin, Iran [17] and considered as the second center of origin and diversification [24]. However, it is still a minor and ignored fruit crop in Pakistan with a few registered cultivars [11]. Moreover, apart from diversification, regional/local consumption patterns, and great market demand for pomegranate in Pakistan, the detection of organic acids and soluble sugars as quality parameters had not yet been documented. In addition, the correlation among different landraces of pomegranate and its biochemical quality attributes needs to be explored underpinning the selection of elite accessions in the juice industry to reduce malnutrition and ensure food security in the region, which in the present study constitutes the novelty. Therefore, an attempt was undertaken in the present investigation to detect and identify varying amounts of soluble sugars and organic acids in 23 pomegranate genotypes using high performance liquid chromatography (HPLC), which would help to screen out the best genotypes for the fresh and juice industry to reduce malnutrition in human beings and ensure to select these elite accessions in the variety improvement program.

## 2. Results

### 2.1. Characterization of Acids and Sugars in Fruits of 23 Pomegranate Genotypes

Significantly high variation was recorded among the 11 detected acids in 23 pomegranate landraces under HPLC analysis. Citric acid was the main acid, followed by succinic and malic acids, however, other acids were in traces and or not detected in some genotypes (Table 1). The range of citric, succinic, lactic, malonic methylmalonic, pyruvic, and fumaric acids were 1.81–65.88%, 0.00–34.45%, 0.00–39.37%, 0.0–54.63%, 0.00%–20.68%, 0.00–17.76%, and 0.00–24.88%, respectively. Sour cultivated pomegranate (KK-I) had the highest quantity of acids followed by MSA-II, Q-II, and RN-I. Moreover, KK-I had a high level of malonic (54.70%) followed by citric and succinic acids, however, a sweet–sour landrace of Multan region (MSA-II) had a high concentration of lactic acid followed by fumaric and pyruvic acids. Citric acid was the principal acid in all wild and cultivated sour genotypes (Q-II, KK-I, MAN-VI, and MK-IV), whereas, malonic, methylmalonic, and fumaric acids were noticed in traces in numerous genotypes and not sensed in others (Table 1). Traces of tartaric, oxalic, oxaloacetic, lactic, malonic, methylmalonic, pyruvic, and fumaric acids were noted in wild landraces with no malonic, methylmalonic, and fumaric acids in AK-I, AM-X, and AG-XIII. Oxalic and lactic acids were not perceived in RLF-I and CK-II, while malonic acid was not detected in MG-II, MK-III, and Q-II (Table 1). Sourness index (SI) was 35.55%, 18.88% and 18.13% in RLF-II, MAN-IV, and RLF-I, respectively and grouped as sweet genotypes. Commercial genotypes (Q-I, MK-III, and MSA-II) grouped as sweet–sour with SI ranged from 6% to 12%, whereas, non-commercial landraces (BR-I, DH-II, RLF-VI, CK-I, CK-II, and CB-I) grouped as sour–sweet with SI ≤ 1. All wild and some cultivated pomegranates (MAN-VI, KK-I, MG-II, MK-IV, and Q-II) were grouped as sour for SI value ≤ 0.50 (Table 1).

Variability in eight detected sugars was significantly high in 23 pomegranate landraces with highest concentration of fructose followed by glucose and d-arabinose, however, MAN-IV (a commercial accession) had high sugar contents followed by DH-II, Q-I, and RLF-I, whereas, traces of sugars were observed in sour and all wild landraces (Table 1). The range of fructose, glucose, d-arabinose, maltose, and mannose was 0.28%–5.47%, 0.44%–2.76%, 1.78%–4.42%, 0.16%–2.87%, and 0.39%–3.79%, respectively. Among wild genotypes, a landrace (MT-III) naturally growing in Murree hills produced a high quantity of fructose and d-arabinose, whereas an Abbottabad accession (AM-X) showed a high level of melazitose and RN-I (accession from Rawalakot) had produced a high level of glucose.

High variation was recorded in fruit weight, aril weight, and wood portion index (WPI) in wild and cultivated pomegranate genotypes. There was a high range in fruit weight 1.29–247.42 g, 100 arils weight 10.99–113.78 g, and WPI 2.39%–17.25%. Commercial genotypes of Tarnab (TG) and Rahim Yar Khan (RLF-I and RLF-II) had the highest fruit weight (247) and (245 and 234 g), respectively. The least fruit weight was recorded in wild and some non-commercial domesticated landraces (CB-I, CK-II, and CK-I). The 100 arils weight was highest in TG followed by MK-IV, MK-III, and RLF-II with 113, 68, 61, and 58 g, respectively. Minimum aril weight was recorded in wild landraces followed by Q-II and CB-I (cultivated landraces). In general observation, fruit weight is directly associated with aril weight, however, an accession, commercially grown in Quetta (Q-II) was less juicy with minimum aril weight (15.12 g) as compared to fruit weight of 219.92 g (Table 2). The wood portion index (WPI) ranged from 2.39% to 17.25% in commercial and wild landraces. All wild and some non-commercial landraces (CK-I, BR-I, and DH-II) had high WPI. However, all commercial genotypes had low WPI, however, a commercial accession from Multan region (MSA-II) had high WPI (14.59%).

### 2.2. Correlation of Traits and Association of Genotypes with Traits in PCA Biplot

**Figure 1:**
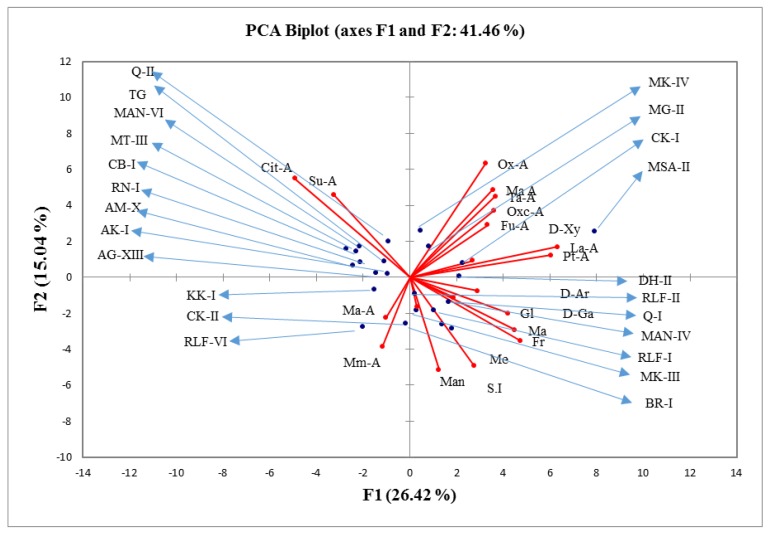
Pomegranate landraces association with fruit traits in a principal component analysis (PCA) biplot. Fru; fructose, Glu; glucose, Mal: maltose, Mel: melezitose, Man: mannose, d-Xy: d-xylose, d-Ar: d-arabinose, d-Ga: d-galactose, Cit-A: citric acid, Ma-A: malic acid, Su-A: succinic acid, Ta-A: tartaric acid, Ox-A: oxalic acid, Oxc-A: Oxaloacetic acid, La-A: lactic acid, Ma-A: malonic acid, Mm-A: methylmalonic acid, Pt-A: pyruvic acid, Fu-A: fumaric acid, SI: sourness index.

Pearson correlation analysis showed a strong correlation between lactic acid and pyruvic acid (r = 0.92). Similarly, tartaric and oxalic acid showed a strong correlation (*r* = 0.84) at *p* ≤ 0.05, whereas, citric acid had a negative correlation (*r* = −0.70) with sugar contents (Table 3). Pyruvic and lactic acid had a moderate correlation with glucose and mannose (*r* = 0.57 and *r* = 0.57), respectively. Among sugars, fructose, and glucose showed a moderate correlation (*r* = 0.62). The moderate negative correlation of *r* = −0.69 was recorded between the citric acid and sourness index (SI). In the PCA (Principal component analysis) biplot, scattering of pomegranate genotypes and HPLC sugars-acids in all planes proved high diversity in Pakistani pomegranates. Cultivated genotypes (MK-IV, MG-II, and MSA-II) had high bonding with oxalic acid, malic acid, and d-xylose, respectively. Citric and succinic acids were recorded high in nine selected genotypes with two commercial cultivars of Quetta- (Q-II) and Tarnab-Gulabi (TG). Small-size low-quality fruits of Khushab-I, Chakwal-II, and RLF-VI were enriched in malic and methylmalonic acids. Commercial varieties of Balochistan (MK-III and Q-I) had an excellent association with fructose and d-galactose, however, commercial cultivar (RLF-I) had high contents of glucose as compare to rest of commercial pomegranate genotypes (Figure 1).

### 2.3. Phylogenetic Relation of Pomegranate Landraces Based on Sugar and Acid Profiling

**Figure 2:**
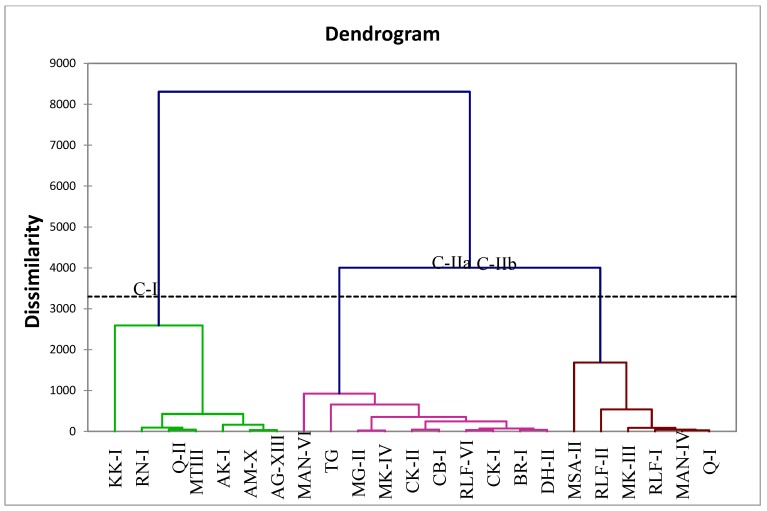
UPGMA (unweighted pair group method with arithmetic mean) dendrogram of pomegranate landraces for organic acids and sugars.

All wild and cultivated pomegranates successfully grouped into two main classes separately with 43.77% of total variability within classes and 56.23% between classes (Figure 2). Class (C-I) consisted of wild (5) and two sour cultivated pomegranates with 71.36% similarity, however, a Kanhatti garden accession (KK-I) not clustered with any of other landraces, showing a high level of dissimilarity from the rest of the germplasm. Cultivated pomegranates in class-II grouped into two subclasses with 10 genotypes in C-IIa with maximum diversity (51.36%) in acids and sugar contents. However, C-IIb had only six landraces with 60.12% of total variability as shown in Figure 2.

## 3. Discussion

There was a high variation in morphochemical traits of pomegranate germplasm with a high concentration of citric and malic acids in sour and sweet landraces, respectively, indicating that sourness is controlled by citric acid in the present study and similar results were confirmed [19]. Significantly high sugar contents in cultivated genotypes might be due to environment and continuous selection and breeding, thus change in genetics [25,26,27]. Moreover, high fruit and aril weight genotypes of Quetta, Mastung, Chakwal, Muzaffargarh, and Rahim Yar Khan were sweet and sweet–sour depicting that these might be developed from sour genotypes through breeding, adaptability and cultural practices. Underweight landraces (wild and non-commercial) had high WPI with low juiciness (less aril weight), which could be improved through subsequent crosses with less WPI genotypes. A high level of diversity was assessed in Pakistani pomegranate germplasm on the basis of biochemical characters like sugars, phenolics, and antioxidants by Nafees et al. [11]. However, Tunisian and Assaria pomegranate cultivars were characterized on the basis of malic and oxalic acids by Miguel et al. [20]. Fructose was predominant in sugars followed by glucose in selected landraces (wild and cultivated) in the current situation, which shows an agreement with the findings of Hasnaoui et al. [10] who reported 53.9% and 43.4% fructose and glucose, respectively, whereas different results were recorded in pomegranate fruit samples of Turkey, Russia, and Saudi Arabia with glucose as an abundant sugar [15,28,29]. The findings of the present study are in line with Akbarpour et al. [13] who reported higher sugar contents in Spanish pomegranate while traces of sugars were reported in sour genotypes (wild and cultivated) of Tunisia, Turkey, and Persia [29,30]. Akbarpour et al. [13] recorded 103.38–505 g and 196–674 g fruit weight in Iranian and Tunisian wild and cultivated pomegranates, respectively, whereas, wild and domesticated pomegranates of Pakistan are low in fruit weight with high WPI, which might be attributed to a lack of modern production technology and less diverse germplasm as compared to the world pomegranate repository. These results are in line with the findings of [16,27,28] for low fruit weight and high WPI in sour pomegranates of Tunisia, Spain, and Iran.

We found an antagonistic and parallel correlation between citric acid and fructose; and lactic acid and sugar contents, respectively. This might be because these traits are interlinked with each other especially at the fruit ripening stage. These results are substantiated with the findings of Hasnaoui et al. [10] in Tunisian commercial pomegranates. Cultivated pomegranates showed significantly high variation as compared to wild (sour) accessions, which might be due to more diverse geographic conditions in growing regions, as this is also described by Hasnaoui et al. [10] and reported a high level of variation in acid and sugar contents in cultivated pomegranates as compared to wild. Moreover, a significantly high correlation of citric acid with sour pomegranates and glucose contents with sweet genotypes was advocated [10].

Hierarchical ascendant classification successfully grouped all wild and two cultivated pomegranates into the same class, however, other cultivated genotypes were grouped together independent of the growing region and thus there is no clarity for the role of geographic conditions, which could otherwise be stated after a complete molecular analysis in future studies of these accessions. The findings of Miguel et al. [20] in Tunisian pomegranates are in line with the clustering of our genotypes, which clustered describing no direct effect of geographic conditions. Overlapping of landraces of different regions in the dendrogram is evidence that the germplasm sharing occurred, which is also substantiated by [9,31] in Pakistani, Tunisian, and Iranian pomegranates. This shows that although there are some morphological differences in fruit traits, yet these landraces may probably be the mutants of each other [32]. Moreover, molecular characterization proved similarities in morphological, biochemical, and DNA studies with a successful grouping of genotypes in dendrogram analysis [33,34,35,36]. A wide range of diversity in sugars and organic acids indicated that Pakistan had a diverse gene pole of pomegranate, which elaborate the potential in variety improvement to strengthen the pomegranate industry in the country, which could help to reduce malnutrition and food security issues in the region.

## 4. Materials and Methods

### 4.1. Collection of Fruit Samples and Preparation For Characterization

Fruit samples were collected from pomegranate plants of 23 selected accessions growing in arid (Bahawalpur, D. G. Khan, Multan, Pakistan), semiarid (Chakwal, Pakistan), hot temperate (Quetta, Pakistan), and cold temperate regions of Khyber Pakhtunkhwa and Rawalakot [37] on sandy loam to clay loam and silt loam soils of Pakistan [38]. Harvesting was done at the full fruit color break stage with TSS ≥ 15° Brix and after a thorough discussion with growers as to when commercial fruit harvesting start, moreover, harvesting time of certain accessions was different in various regions. Various qualitative characters of selected genotypes and the geographic conditions (GPS) of growing regions are described in (Table 4).

Five pomegranate plants were selected for each accession line and five fruits were randomly harvested from each plant to record fruit and 100 arils weighed on digital weighing balance (Ohaus Scout SPX2201 Digital Scale, WA, USA) in Plant Tissue Culture Laboratory, Institute of Horticultural Sciences (IHS), University of Agriculture Faisalabad (UAF) Pakistan, whereas, HPLC analysis for sugars and organic acids was performed in High Tech. Lab. UAF. In this regard, aril samples (0.5 g) were homogenized in a pestle mortar and dissolved in 2.5 mL d_3_H_2_O, centrifuged (Eppendorf 3810 R, Hamburg, Germany) at 10,000 rpm at 4 °C for 10 min as previously described Razzaq et al. [39], the supernatant was kept at temperature of −18 °C for further analysis.

### 4.2. HPLC Analysis

The standards of sugars (glucose, fructose, galactose, arabinose, maltose, mannose, melezitose, and xylose) and organic acids (citric, malic, succinic, tartaric, oxalic, oxaloacetic, lactic, methylmalonic, malonic, pyruvic, and fumaric acids) were purchased from Sigma-aldrich Chemicals Inc. Shangai, China). One milliliter of the centrifuged liquid of the aril stock solution was filtered through a 0.45 µm Millipore size membrane filter and then injected into HPLC (LC-10A HPLC Series, Shimadzu, Kyoto, Japan). The HPLC equipped with a pump system, a refractive index detector (RID-10A) for sugar analysis at 214 nm, and a UV/Vis detector (SPD-20A) monitored at 210 nm, for the analysis of organic acids. Identification and quantification of acids were done by injecting 20 µL stock solution to separate different acids on a Supelcogel^TM^ C-610H column (30 cm × 7.8 mm, i.e., Supelco, Bellefonte, PA, USA) by using 0.1% phosphoric acid as a mobile phase at a flow rate of 0.6 mL min^−1^ in the isocratic mode of HPLC at room temperature (26 °C).

Whereas, the Booij et al.’s method [27] was used to detect sugars by inserting 20 µL aril stock solution in refractive index detector, performed on a μ Bondapak-NH2 column (30 cm × 3.9 mm, i.e., water, Milford, MA, USA) at 214 nm using acetonitrile/water (85:15, *v*/*v*) as the mobile phase at room temperature (26 °C). Identified sugars were quantified on the bases of peak areas of external standards consisting of glucose (1%), fructose (1%), sucrose (1%), and other sugar solutions.

### 4.3. Sourness Index (SI)

Selected pomegranates were classified as sweet, sweet–sour, sour–sweet, and sour by calculating the ratio of total soluble sugars to citric acid concentrations following the method given in Hasnaoui et al. [10].

### 4.4. Statistical Analyses

Genotypes were considered as treatments (23) with five replications of plants within each accession. Data was analyzed in statistical software (Statistics 8.1) under CRD at *p* ≤ 0.05. Moreover, data represent the mean of replicated data ± standard deviation. Standardized data were analyzed to determine the correlation between selected fruit attributes using Pearson correlation, cluster analysis to obtain hierarchical association using the Euclidean distance method as a dis-similarity measure and principal component analysis done using the excel software (XLSTAT 2014 5 version). The significance was set at *p* ≤ 0.05 for comparing the means by using (Tukey–Kramer test).

## 5. Conclusions

To situate the problem addressed here, it was found that citric acid, succinic acid, fructose, and glucose were abundant among the detected acids and sugars in selected wild and cultivated pomegranates in local conditions of Pakistan. Citric and malic were the main acids in controlling the sourness and sweetness, respectively. These results were further verified by cluster analysis, which showed that the morphometric composition of pomegranates was predominately determined by cultivars under the influence of growing regions. The genotypes (RLF-II, MAN-IV, RLF-I, and TG) could have good market demand for high fruit weight, juiciness, and sweetness. However, Q-I, MK-III, and MSA-II were grouped as sweet–sour with a good juice percentage but less market attention. Moreover, good acid profiling in wild and non-commercial genotypes can excel in the juice-based food industry and need successive crosses for variety improvement.

## Figures and Tables

**Table 1:** Identification and quantification of tested organic acids in fruits of 23 pomegranate landraces.

Land. Races	Citric-Acid*	Malic-A*	Succinic A*	Tar-A*	Oxalic-A*	Oxalo-A*	Lactic-A*	M Mel-A*	Mal-A*	Pyruvic-A*	Fumaric-A*	S.I.
BR-I	25.29 ± 1.4	0.72 ± 0.02	0.00 ± 0.00	0.68 ± 0.04	0.93 ± 0.7	0.95 ± 0.8	6.61 ± 1.15	0.07 ± 0.12	1.32 ± 0.03	3.14 ± 0.04	1.45 ± 0.04	0.64 ± 0.04
DH-II	23.92 ± 1.59	2.37 ± 0.04	0.00 ± 0.00	0.67 ± 0.05	2.83 ± 0.09	2.80 ± 0.1	13.77 ± 1.43	0.00 ± 0.00	0.58 ± 0.04	6.46 ± 0.27	0.86 ± 0.06	0.66 ± 0.04
MAN-IV	1.81 ± 0.89	0.48 ± 0.04	3.56 ± 0.02	1.33 ± 0.04	0.60 ± 0.08	0.59 ± 0.02	5.92 ± 1.71	1.29 ± 0.06	4.79 ± 0.09	2.80 ± 0.03	0.32 ± 0.05	18.88 ± 0.18
MAN-VI	38.50 ± 0.77	0.29 ± 0.02	0.09 ± 0.07	1.02 ± 0.03	0.58 ± 0.03	0.61 ± 0.06	2.59 ± 1.03	20.68 ± 0.41	24.60 ± 0.1	1.87 ± 0.04	0.14 ± 0.03	0.45 ± 0.04
RLF-I	1.69 ± 0,61	1.66 ± 0.05	3.63 ± 0.07	1.72 ± 0.05	0.00 ± 0.00	1.99 ± 0.04	11.63 ± 2.09	1.81 ± 0.03	5.33 ± 0.06	5.33 ± 0.07	0.27 ± 0.03	18.13 ± 0.47
RLF-II	1.33 ± 1,0	1.07 ± 0.06	6.35 ± 0.11	2.86 ± 0.06	2.58 ± 0.04	2.23 ± 0.05	17.63 ± 3.61	2.91 ± 0.05	16.68 ± 0.16	3.32 ± 0.05	0.65 ± 0.04	35.55 ± 0.30
RLF-VI	21.75 ± 1.45	0.62 ± 0.05	5.69 ± 0.16	1.75 ± 0.07	0.74 ± 0.05	0.77 ± 0.06	12.51 ± 2.04	0.58 ± 0.03	6.81 ± 0.08	4.70 ± 0.11	0.00 ± 0.00	0.77 ± 0.03
MSA-II	3.24 ± 0.74	3.34 ± 0.09	5.83 ± 0.12	4.58 ± 0.04	4.09 ± 0.07	3.97 ± 0.05	39.37 ± 0.65	0.00 ± 0.00	0.13 ± 0.02	17.76 ± 0.22	24.88 ± 0.18	6.67 ± 0.14
CK-I	22.9 ± 1.46	3.98 ± 0.09	7.36 ± 0.05	2.14 ± 0.02	1.93 ± 0.04	4.76 ± 0.11	9.18 ± 0.07	3.26 ± 0.15	3.87 ± 0.04	4.55 ± 0.07	0.00 ± 0.00	0.61 ± 0.06
CK-II	28.51 ± 1.98	0.88 ± 0.08	10.47 ± 0.73	1.072 ± 0.04	0.98 ± 0.04	0.82 ± 0.07	0.00 ± 0.00	1.44 ± 0.04	5.88 ± 0.05	1.74 ± 0.04	0.00 ± 0.00	0.56 ± 0.05
CB-I	27.67 ± 1.56	2.16 ± 0.03	12.43 ± 1.94	0.97 ± 0.03	1.29 ± 0.05	2.52 ± 0.05	6.71 ± 0.08	1.89 ± 0.03	11.56 ± 0.07	0.58 ± 0.04	0.00 ± 0.00	0.65 ± 0.10
KK-I	56.42 ± 0.96	1.28 ± 0.04	24.63 ± 0.50	0.96 ± 0.03	0.84 ± 0.06	1.47 ± 0.5	7.04 ± 0.07	1.01 ± 0.02	54.63 ± 0.22	1.78 ± 0.03	0.07 ± 0.06	0.23 ± 0.05
MG-II	37.84 ± 1.75	0.63 ± 0.08	3.85 ± 0.12	4.90 ± 0.06	4.44 ± 0.07	4.22 ± 0.5	7.13 ± 0.03	0.00 ± 0.00	0.00 ± 0.00	0.85 ± 0.06	2.40 ± 0.03	0.37 ± 0.06
MK-III	2.28 ± 0.83	0.57 ± 0.18	9.82 ± 0.64	1.07 ± 0.04	0.52 ± 0.04	0.49 ± 0.03	3.94 ± 0.07	1.15 ± 0.05	0.00 ± 0.00	0.86 ± 0.99	0.31 ± 0.01	11.47 ± 0.35
MK-IV	38.85 ± 1.17	3.59 ± 0.34	5.18 ± 0.15	5.15 ± 0.05	4.69 ± 0.09	0.07 ± 0.06	8.89 ± 0.01	0.00 ± 0.00	0.23 ± 0.02	0.60 ± 0.09	0.14 ± 0.02	0.41 ± 0.04
TG	22.18 ± 1.12	0.82 ± 0.09	12.29 ± 0.38	1.18 ± 0.02	1.06 ± 0.05	0.85 ± 0.04	1.54 ± 0.02	0.90 ± 0.06	0.06 ± 0.06	1.66 ± 0.05	24.51 ± 0.1	0.71 ± 0.04
Q-I	2.10 ± 0.79	0.71 ± 0.14	4.06 ± 0.05	2.46 ± 0.02	0.86 ± 0.06	0.85 ± 0.02	6.92 ± 0.04	0.93 ± 0.01	1.58 ± 0.04	1.88 ± 0.03	0.00 ± 0.00	12.94 ± 0.25
Q-II	53.49 ± 0.81	1.73 ± 0.06	34.45 ± 0.48	2.20 ± 0.08	2.07 ± 0.04	1.99 ± 0.03	2.90 ± 0.03	0.23 ± 0.04	0.00 ± 0.00	2.48 ± 0.07	0.00 ± 0.00	0.25 ± 0.03
AK-I	58.37 ± 1.04	0.41 ± 0.16	4.86 ± 0.18	1.47 ± 0.04	1.20 ± 0.09	1.13 ± 0.08	6.16 ± 0.04	0.62 ± 0.02	0.00 ± 0.00	1.34 ± 0.05	0.75 ± 0.05	0.16 ± 0.02
AM-X	46.08 ± 1.85	1.85 ± 0.26	18.88 ± 0.48	1.48 ± 0.04	1.29 ± 0.07	1.86 ± 0.05	3.28 ± 0.06	0.00 ± 0.00	0.06 ± 0.06	1.05 ± 0.06	0.00 ± 0.00	0.24 ± 0.04
AG-XIII	53.33 ± 1.72	0.81 ± 0.36	14.68 ± 0.39	1.81 ± 0.08	2.28 ± 0.03	1.27 ± 0.05	3.35 ± 0.05	1.96 ± 0.06	0.54 ± 0.04	1.78 ± 0.04	0.00 ± 0.00	0.20 ± 0.02
MT-III	54.93 ± 1.59	2.05 ± 0.70	26.09 ± 0.08	1.33 ± 0.06	1.21 ± 0.04	1.62 ± 0.04	4.24 ± 0.04	0.00 ± 0.00	0.13 ± 0.02	0.91 ± 0.04	0.85 ± 0.06	0.17 ± 0.02
RN-I	65.88 ± 1.14	2.33 ± 0.78	25.62 ± 0.39	1.13 ± 0.04	1.11 ± 0.09	1.84 ± 0.05	2.12 ± 0.03	0.08 ± 0.07	0.31 ± 0.03	0.83 ± 0.07	0.63 ± 0.03	0.15 ± 0.02

Abbreviations: A*; Acid; Tar; Tartaric; Oxal A*; Oxaloacetic acid; M Mel; Methylmalonic acid; Mal; Malonic acid; S.I.; Sourness index. All the data presented are the mean ± SE of three replications with significant differences at *p* ≤ 0.05 (Tukey–Kramer test).).

**Table 2:** Identification and quantification of various sugars in HPLC and selective fruit trait in 23 pomegranate landraces.

Landraces	Fructose	Glucose	Maltose	Mannose	Melezitose	d-Xylose	d-Arabinose	d-Galactose	Fruit Weight (g)	aril Weight (g)	WPI
BR-I	**3.89 ± 0.09**	**3.58 ± 0.04**	**2.87 ± 0.04**	1.80 ± 0.02	**2.21 ± 0.08**	0.79 ± 0.03	0.88 ± 0.06	0.07 ± 0.18	120.72 ± 10.61	36.19 ± 1.94	**9.71 ± 0.81**
DH-II	**3.87 ± 0.10**	3.02 ± 0.04	1.90 ± 0.03	1.12 ± 0.02	**2.78 ± 0.02**	1.76 ± 0.05	**2.14 ± 0.07**	1.23 ± 0.03	101.11 ± 13.82	33.46 ± 3.81	**7.27 ± 0.57**
MAN-IV	**4.84 ± 0.06**	3.86 ± 0.10	1.13 ± 0.07	0.91 ± 0.02	1.54 ± 0.08	**1.96 ± 0.07**	1.91 ± 0.08	**3.27 ± 0.05**	164.27 ± 5.06	41.09 ± 0.68	5.64 ± 0.89
MAN-VI	2.80 ± 0.16	2.42 ± 0.10	1.35 ± 0.03	2.33 ± 0.03	1.32 ± 0.05	0.91 ± 0.09	1.11 ± 0.10	1.64 ± 0.07	164.27 ± 5.06	41.09 ± 0.68	5.64 ± 0.89
RLF-I	**4.88 ± 0.11**	2.57 ± 0.07	**2.34 ± 0.04**	**3.79 ± 0.02**	0.07 ± 0.02	0.36 ± 0.07	1.68 ± 0.14	**1.89 ± 0.08**	**245.22 ± 12.22**	**53.35 ± 1.32**	4.05 ± 0.31
RLF-II	**5.03 ± 0.17**	2.14 ± 0.07	1.79 ± 0.03	**2.06 ± 0.05**	1.09 ± 0.04	1.49 ± 0.05	**2.14 ± 0.03**	0.32 ± 0.04	**234.8 ± 12.66**	**58.80 ± 2.25**	4.45 ± 0.24
RLF-VI	3.06 ± 0.04	2.98 ± 0.03	1.87 ± 0.05	0.95 ± 0.05	1.82 ± 0.06	0.88 ± 0.09	**2.76 ± 0.04**	0.33 ± 0.04	125.52 ± 8.52	44.03 ± 1.61	**7.22 ± 0.25**
MSA-II	**3.03 ± 0.07**	**4.42 ± 0.10**	**2.33 ± 0.01**	0.90 ± 0.03	0.66 ± 0.07	1.59 ± 0.07	2.1 ± 0.08	**1.83 ± 0.06**	126.03 ± 6.44	**52.89 ± 2.24**	**14.59 ± 0.43**
CK-I	**2.03 ± 0.06**	3.31 ± 0.10	1.13 ± 0.09	1.80 ± 0.04	0.87 ± 0.04	1.26 ± 0.05	2.11 ± 0.06	1.43 ± 0.05	77.19 ± 7.00	31.56 ± 1.72	**9.69 ± 0.31**
CK-II	2.77 ± 0.11	3.21 ± 0.07	1.10 ± 0.06	1.80 ± 0.02	0.38 ± 0.03	0.91 ± 0.09	1.02 ± 0.04	1.09 ± 0.02	60.61 ± 8.19	31.56 ± 2.26	3.81 ± 0.11
CB-I	2.15 ± 0.10	2.89 ± 0.06	1.23 ± 0.02	0.87 ± 0.05	1.23 ± 0.03	0.00 ± 0.00	1.83 ± 0.05	**1.89 ± 0.09**	39.53 ± 1.48	18.68 ± 0.65	4.32 ± 0.27
KK-I	2.93 ± 0.05	2.98 ± 0.04	1.06 ± 0.03	0.89 ± 0.01	0.81 ± 0.02	**1.87 ± 0.05**	2.15 ± 0.05	0.87 ± 0.04	160.95 ± 6.74	**54.67 ± 1.74**	4.14 ± 0.22
MG-II	2.14 ± 0.03	2.88 ± 0.06	1.21 ± 0.03	**3.06 ± 0.04**	1.3 ± 0.08	**1.87 ± 0.08**	0.76 ± 0.09	1.34 ± 0.03	196.17 ± 10.49	44.34 ± 2.68	4.58 ± 0.25
MK-III	**4.79 ± 0.08**	**3.25 ± 0.03**	1.22 ± 0.06	**2.86 ± 0.03**	0.94 ± 0.06	1.36 ± 0.06	1.02 ± 0.02	1.25 ± 0.04	**218.76 ± 14.51**	**61.97 ± 3.85**	4.24 ± 0.26
MK-IV	2.78 ± 0.03	1.78 ± 0.18	2.15 ± 0.4	0.77 ± 0.04	1.12 ± 0.07	1.09 ± 0.08	1.13 ± 0.03	1.05 ± 0.04	113.85 ± 5.59	**68.52 ± 4.43**	3.49 ± 0.40
TG	2.90 ± 0.09	2.07 ± 0.13	0.34 ± 0.06	0.39 ± 0.01	1.09 ± 0.09	1.52 ± 0.07	1.89 ± 0.09	0.87 ± 0.04	**247.42 ± 17.52**	**113.78 ± 6.65**	2.39 ± 0.29
Q-I	**5.37 ± 0.03**	3.11 ± 0.10	1.72 ± 0.05	1.15 ± 0.06	**2.04 ± 0.06**	1.73 ± 0.09	1.33 ± 0.06	0.81 ± 0.03	**218.33 ± 12.72**	**46.89 ± 3.67**	3.52 ± 0.23
Q-II	**3.46 ± 0.02**	2.75 ± 0.09	1.24 ± 0.02	0.54 ± 0.05	1.16 ± 0.04	**2.11 ± 0.09**	1.12 ± 0.03	1.79 ± 0.01	**219.92 ± 14.75**	15.12 ± 0.78	3.71 ± 0.41
AK-I	1.28 ± 0.04	2.07 ± 0.05	1.02 ± 0.01	0.81 ± 0.05	1.07 ± 0.04	0.66 ± 0.07	1.09 ± 0.09	1.22 ± 0.04	41.16 ± 3.75	12.34 ± 0.79	**17.25 ± 0.94**
AM-X	2.20 ± 0.10	1.81 ± 0.03	0.91 ± 0.07	1.89 ± 0.08	**2.02 ± 0.04**	0.74 ± 0.07	1.68 ± 0.08	0.29 ± 0.01	45.04 ± 4.35	11.39 ± 0.89	**11.03 ± 0.82**
AG-XIII	1.82 ± 0.04	2.67 ± 0.06	0.16 ± 0.03	0.64 ± 0.06	0.72 ± 0.04	1.07 ± 0.08	0.89 ± 0.02	0.96 ± 0.04	34.29 ± 1.72	18.58 ± 0.76	**16.75 ± 0.58**
MT-III	2.26 ± 0.04	1.89 ± 0.05	0.72 ± 0.02	0.44 ± 0.19	0.17 ± 0.05	0.90 ± 0.07	**2.19 ± 0.06**	0.87 ± 0.05	28.02 ± 8.48	10.99 ± 1.92	**15.21 ± 0.81**
RN-I	2.033 ± 0.07	2.89 ± 0.09	0.16 ± 0.02	0.84 ± 0.09	1.07 ± 0.03	0.78 ± 0.06	0.44 ± 0.03	1.05 ± 0.05	15.82 ± 2.50	11.91 ± 1.74	**14.36 ± 0.55**

Abbreviation: WPI (wood portion index). All the data presented are the mean ± SE of three replications with significant differences at *p* ≤ 0.05 (Tukey–Kramer test). Values in bold letters are highest means of different sugars of 23 landraces measured in Brix unit (%).

**Table 3:** Pearson correlation between acid and sugar contents detected in selected pomegranate landraces based in Pakistan.

Landraces	Fru.	Glu.	Mal.	Man.	Mel.	d-Xy	d-Ar.	d-Gl.	Cit-A	Ma A	Su-A	Ta-A	Ox-A	Oxc-A	La-A	Mm-A	Ma-A	Pt.-A	Fu-A	S.I
Fr	**1**																			
Gl	**0.62**	**1**																		
Ma	0.45	0.32	**1**																	
Man	0.23	0.09	0.35	**1**																
Me	0.05	0.09	0.26	−0.11	**1**															
d-Xy	0.36	0.32	0.01	−0.12	0.21	**1**														
d-A	0.29	0.09	0.21	−0.19	0.06	0.13	**1**													
d-G	0.31	0.36	−0.07	0.04	−0.25	0.17	0.03	**1**												
Cit-A	**−0.7**	−0.45	**−0.55**	−0.41	−0.12	−0.22	−0.36	−0.22	**1**											
Ma A	0.3	0.08	0.26	−0.16	−0.1	−0.01	0.25	0.13	−0.02	**1**										
Su-A	−0.26	−0.25	**−0.58**	−0.43	−0.34	0.05	−0.07	−0.08	**0.62**	0.04	**1**									
Ta-A	0.04	0	0.28	0.07	−0.17	0.31	−0.08	0.05	−0.13	0.44	−0.18	**1**								
Ox-A	−0.13	−0.01	0.2	−0.12	0.05	0.35	−0.06	−0.02	0.06	**0.54**	−0.12	**0.84**	**1**							
Oxc-A	0.19	0.29	0.05	0.18	−0.09	0.16	0.21	0.15	−0.05	**0.52**	0.01	0.35	0.47	**1**						
La-A	0.4	0.47	**0.57**	0.02	−0.04	0.22	0.43	0.13	−0.45	0.5	−0.32	**0.51**	0.49	0.49	**1**					
Mm-A	−0.08	−0.13	−0.03	0.27	−0.02	−0.14	−0.09	0.19	0	−0.21	−0.28	−0.18	−0.23	−0.16	−0.16	**1**				
Ma-A	−0.13	0.01	−0.01	0.09	−0.11	0.12	0.27	−0.03	0.12	−0.13	0.13	−0.22	−0.23	−0.08	−0.04	0.38	**1**			
Pt-A	0.48	**0.57**	0.49	−0.02	−0.05	0.22	0.42	0.19	−0.42	0.44	−0.27	0.32	0.33	0.47	**0.92**	−0.07	−0.09	**1**		
Fu-A	0.15	0.2	0.01	−0.23	−0.14	0.23	0.2	0.06	−0.26	0.17	−0.06	0.27	0.26	0.21	**0.51**	−0.11	−0.16	**0.59**	**1**	
S.I.	0.38	0.09	0.28	0.36	−0.1	0.21	0.24	0.14	**−0.69**	−0.15	−0.28	0.12	−0.1	−0.02	0.28	0.01	0.07	0.15	−0.05	**1**

Abbreviation: Fru: Fructose, Glu: Glucose, Mal: Maltose, Man: Manose, Mel: Melezitose, d-Xy: d-Xylose, d-Ar: d-Arabinose, d-Ga: d-Galactose, Cit-A: Citric acid, Ma-A: Malic acid, Su-: Succinic acid, Ta-A-: Tartaric acid, Ox-A: Oxalic acid, Oxc- A: Oxaloacetic acid, La-A: Lactic acid, Mm-A: Methylmalonic acid, Ma-A: Malonic acid, Pt-A: Pyruvic acid, Fu-A: Fumaric acid, SI: Sourness index. Values in bold are significantly different at *p* ≤ 0.05 (Tukey–Kramer test).

**Table 4:** Detail of accessions characters, code, and recorded GPS data of their growing regions.

S. No.	Districts-Sites	Accessions Characters and Their Allotted Codes	Latitude (N)	Longitude (E)	Elevation (m.a.s.l)
1	D.G. Khan	Small greenish red fruit with white arils (DH-I)	70°63	30°05	90.52
2	BWP-RARI	Small greenish fruit, pinkish sour arils (BR-I)	73°27	29°98	119.48
3	Muzafar-Garh	Medium oblong fruit, white arils (MAN-IV)	70°91	29°38	102.99
4		Pinkish fruit, white pink sour arils (MAN-IV)			
5	R.Y. Khan Liaqatpur	Pinkish fruit, white pink sweet arils (RFL-I)	70°57	28°55	95.09
6		Greenish large fruit, white sweet arils (RFL-II)			
7		Small red fruit, red white sour arils (RFL-VI)			
8	Multan	Large greenish fruit, white arils (MSA-II)	71°29	29°88	151.99
9	Chakwal Kallar kahar	Small round red fruit with white arils (CK-I)	72°42	32°47	634.89
10		Small round red fruit with red arils (CK-II			
11		Small greenish fruit, pinkish arils (CB-I)			
12	HRS-Khushab	Small greenish fruit with pinkish arils (KK-I)	74°15	36°42	1507.5
13	Quetta Barkhan	Large round with red fruit and arils (Q-I)	71°41	34°	1686.1
14		Large round red fruit with white arils (Q-II)			
15	Mastung Gulab bag Kari Kucha	Large greenish fruit, red pink arils (MG-II)	66°85	29°8	1682.8
16		Large fruit, white soft sweet arils (MK-III)			
17		Greenish fruit, white pinkish arils (MK-IV)			
18	KPK-ARIT	Large red fruit and arils, Tarnab-Gulabi (TG)	71°6	34°25	348.99
19	Abbottabad Kohala Maillot	Small size red fruit with sour red arils (AK-I)	73°18	33°87	1022.6
20		Small size red fruit with red pink arils (AM-X)	73°33	33°89	1202.4
21		Greenish fruit with red arils (AG-XIII)	72°96	33°91	663.55
22	Murree	Small greenish fruit with red arils (MT-III)	73°3	33°84	1335
23	Rawalakot Nakkar	Small size red fruits with red arils (RN-I)	73°34	34°3	1427.6

Abbreviations: D.G khan.: Dera Gazi khan; BWPRARI: Bahawalpur-Regional Agriculture Research Institute; HRS: Horticulture Research Station; KPK-ARIT: Khyber Pakhtunkhwa Agriculture Research Institute, Tarnab; m.a.s.l: meter above sea level.
